# Quantifying health facility service readiness for small and sick newborn care: comparing standards-based and WHO level-2 + scoring for 64 hospitals implementing with NEST360 in Kenya, Malawi, Nigeria, and Tanzania

**DOI:** 10.1186/s12887-024-04578-5

**Published:** 2024-03-12

**Authors:** Rebecca E. Penzias, Christine Bohne, Edith Gicheha, Elizabeth M. Molyneux, David Gathara, Samuel K. Ngwala, Evelyn Zimba, Ekran Rashid, Opeyemi Odedere, Olabisi Dosunmu, Robert Tillya, Josephine Shabani, James H. Cross, Christian Ochieng, Harriet H. Webster, Msandeni Chiume, Queen Dube, John Wainaina, Irabi Kassim, Grace Irimu, Steve Adudans, Femi James, Olukemi Tongo, Veronica Chinyere Ezeaka, Nahya Salim, Honorati Masanja, Maria Oden, Rebecca Richards-Kortum, Tedbabe Hailegabriel, Gagan Gupta, Simon Cousens, Joy E. Lawn, Eric O. Ohuma, with the Health Facility Assessment Data Collection Learning Collaborative Group, Christina Mchoma, Joseph Bilitinyu, Pius Chalamanda, Mirriam Dzinkambani, Ruth Mhango, Fanny Stevens, Joseph Mulungu, Blessings Makhumula, Loveness Banda, Charles Banda, Brian Chumbi, Chifundo Banda, Evelyn Chimombo, Nicodemus Nyasulu, Innocent Ndau, Pilirani Kumwembe, Edna Kerubo, Nyphry Ambuso, Kevin Koech, Noel Waithaka, Calet Wakhungu, Steven Otieno, Felix Bahati, Josphine Ayaga, Jedida Obure, Nellius Nderitu, Violet Mtambo, George Mkude, Mustapha Miraji, Caroline Shayo, Camilius Nambombi, Christopher Cyrilo, Temilade Aderounmu, Akingbehin Wakeel Wale, Odeleye Victoria Yemisi, Akinola Amudalat Dupe, Samuel Awolowo, Ojelabi Oluwaseun A., John Ajiwohwodoma Ovuoraye, Balogun Adeleke Mujaid, Adedoyin Fetuga, Juilana Okanlawon, Flora Awosika, Awotayo Olasupo Michael, Omotayo Adegboyega Abiodun

**Affiliations:** 1 Maternal, Adolescent, Reproductive, & Child Health Centre, London School of Hygiene & Tropical Medicine, London, UK; 2 Rice360 Institute for Global Health Technologies, Rice University, Texas, USA; 3 Ifakara Health Institute, Ifakara, Tanzania; 4 Kamuzu University of Health Sciences (Formerly College of Medicine, University of Malawi), Blantyre, Malawi; 5 School of Global and Public Health, Kamuzu University of Health Sciences, Blantyre, Malawi; 6 Aga Khan University Hospital, Nairobi, Kenya; 7 APIN Public Health Initiatives, Abuja, Nigeria; 8 Department of Paediatrics, Kamuzu Central Hospital, Lilongwe, Malawi; 9 Ministry of Health, Lilongwe, Malawi; 10 Kenya Medical Research Institute (KEMRI)-Wellcome Trust, Nairobi, Kenya; 11 Department of Paediatrics and Child Health, University of Nairobi, Nairobi, Kenya; 12 Academy for Novel Channels in Health and Operations Research (ACANOVA) Africa, Nairobi, Kenya; 13 Newborn Branch, Federal Ministry of Health, Abuja, Nigeria; 14 Department of Paediatrics, College of Medicine, University of Ibadan, Ibadan, Nigeria; 15 Department of Paediatrics, College of Medicine, University of Lagos, Lagos, Nigeria; 16 Department of Paediatrics and Child Health, Muhimbili University of Health and Allied Sciences, Dar Es Salaam, Tanzania; 17 Program Group, Health Programme UNICEF Headquarters, New York, NY USA

**Keywords:** Newborn, Low- and middle-income countries, Inpatient care, Service readiness, Health facility assessment, Small and sick newborn care, Health systems scoring, ENAP coverage targets

## Abstract

**Background:**

Service readiness tools are important for assessing hospital capacity to provide quality small and sick newborn care (SSNC). Lack of summary scoring approaches for SSNC service readiness means we are unable to track national targets such as the *Every Newborn* Action Plan targets.

**Methods:**

A health facility assessment (HFA) tool was co-designed by Newborn Essential Solutions and Technologies (NEST360) and UNICEF with four African governments. Data were collected in 68 NEST360-implementing neonatal units in Kenya, Malawi, Nigeria, and Tanzania (September 2019-March 2021). Two summary scoring approaches were developed: a) standards-based, including items for SSNC service readiness by health system building block (HSBB), and scored on availability and functionality, and b) level-2 + , scoring items on readiness to provide WHO level-2 + clinical interventions. For each scoring approach, scores were aggregated and summarised as a percentage and equally weighted to obtain an overall score by hospital, HSBB, and clinical intervention.

**Results:**

Of 1508 HFA items, 1043 (69%) were included in standards-based and 309 (20%) in level-2 + scoring. Sixty-eight neonatal units across four countries had median standards-based scores of 51% [IQR 48–57%] at baseline, with variation by country: 62% [IQR 59–66%] in Kenya, 49% [IQR 46–51%] in Malawi, 50% [IQR 42–58%] in Nigeria, and 55% [IQR 53–62%] in Tanzania. The lowest scoring was family-centred care [27%, IQR 18–40%] with governance highest scoring [76%, IQR 71–82%]. For level-2 + scores, the overall median score was 41% [IQR 35–51%] with variation by country: 50% [IQR 44–53%] in Kenya, 41% [IQR 35–50%] in Malawi, 33% [IQR 27–37%] in Nigeria, and 41% [IQR 32–52%] in Tanzania. Readiness to provide antibiotics by culture report was the highest-scoring intervention [58%, IQR 50–75%] and neonatal encephalopathy management was the lowest-scoring [21%, IQR 8–42%]. In both methods, overall scores were low (< 50%) for 27 neonatal units in standards-based scoring and 48 neonatal units in level-2 + scoring. No neonatal unit achieved high scores of > 75%.

**Discussion:**

Two scoring approaches reveal gaps in SSNC readiness with no neonatal units achieving high scores (> 75%). Government-led quality improvement teams can use these summary scores to identify areas for health systems change. Future analyses could determine which items are most directly linked with quality SSNC and newborn outcomes.

**Supplementary Information:**

The online version contains supplementary material available at 10.1186/s12887-024-04578-5.

## Key findings

**Table:** 

**1. WHAT WAS KNOWN?**
• Health facility service readiness can be assessed for wider service provision using existing tools (e.g. Service Provision Assessment), but there is a lack of specific tools to measure small and sick newborn care (SSNC), and to assess if a hospital is meeting World Health Organization (WHO) level-2 + SSNC standards. There are a range of methods for scoring service readiness including signal functions or tracer indicators and content-specific scores that can be used to track progress over time and make comparisons between hospitals
• The *Every Newborn* Action Plan (ENAP), launched in 2014, is being implemented by more than 106 countries. The fourth ENAP coverage target is for 80% of districts in every country to have at least one functional level-2 SSNC unit with respiratory support by 2025. However, we found no published scoring methods to quantitatively evaluate progress towards the fourth ENAP target. Currently, progress towards the ENAP coverage target is assessed through country self-report
**2. WHAT WAS DONE THAT IS NEW?**
• NEST360 and UNICEF facilitated co-design of a health facility assessment (HFA) tool for level-2 + SSNC in partnership with four African governments. Level-2 + SSNC includes WHO level-2 interventions, and provision of respiratory support. HFAs were conducted in 68 neonatal units in 64 hospitals in Kenya, Malawi, Nigeria, and Tanzania. We compared two approaches to summarise service readiness for SSNC
• Standards-based scoring: Six health system building blocks (HSBBs), adapted from the WHO framework, assess readiness to provide SSNC according to national and global clinical standards. All items required for SSNC service readiness were included, and scored according to availability and functionality. An overall score was computed by HSBB module and aggregated (equally weighted) for each hospital. Scores were classified as low (<50%), intermediate (50-75%), and high (>75%)
• Meeting criteria for WHO level-2 + scoring: The WHO levels of care include clinical interventions and were adapted to quantify level-2 + care readiness. For each of ten clinical interventions in level-2 + , items required for diagnosis/screening and treatment/management were included and scored. An overall score was computed for each hospital and by clinical intervention. Scores were classified as low (<50%), intermediate (50-75%), and high (>75%)
**3. WHAT WAS FOUND?**
• Standards-based scoring: Of 1508 HFA items, 1043 items or ingredients (69%) for SSNC were scored by availability and functionality. For 68 neonatal units across the four countries, the overall median score was 51% [IQR 48–57%], with some variation by country: 62% [IQR 59–66%] in Kenya, 49% [IQR 46–51%] in Malawi, 50% [IQR 42–58%] in Nigeria, and 55% [IQR 53–62%] in Tanzania. Of the 68 neonatal units, 27 neonatal units had low overall scores of < 50%, and 41 neonatal units had intermediate scores of 50–75%. No neonatal units achieved high scores of > 75%. The lowest scoring HSBB was family-centred care [27%, IQR 18–40%] with governance the highest-scoring HSBB across all countries [76%, IQR 71–82%]. Medical device scores were also low across most hospitals [43%, IQR 38–48%]
• Level-2 + scoring: Of 1508 HFA items, 309 items (20%) for level-2 + SSNC interventions were included. The overall median readiness score for 68 neonatal units was 41% [IQR 35–51%] with some variation by country: 50% [IQR 44–53%] in Kenya, 41% [IQR 35–50%] in Malawi, 33% [IQR 27–37%] in Nigeria, and 41% [IQR 32–52%] in Tanzania. 48 neonatal units had low overall scores of < 50%, and 20 neonatal units had intermediate scores of 50–75%. No neonatal units achieved high scores of > 75%. Overall, readiness to provide antibiotics guided by culture report was the highest scoring intervention [Median 58%, IQR 50–75%] and was the highest scoring intervention in Kenya and Tanzania. Detection and management of neonatal encephalopathy received the lowest score overall and showed the most variability across hospitals [Median 21%, IQR 8–42%]
**4. WHAT NEXT?**
• Data on gaps in service readiness identified by the two scoring approaches can be used by facilities and programmes to select areas for health systems change. These can support government-led quality improvement initiatives to improve care of small and sick newborns. Wider use of this tool and quantification would be more robust than self-report of country progress towards the ENAP coverage target
• Future analyses could determine which items are most directly linked with SSNC quality to help identify a smaller set of items that are most important for tracking health systems gaps

## Background

The World Health Organization (WHO) has organised small and sick newborn care (SSNC) by levels of care with a focus on appropriate, evidence-based clinical interventions [1]. Recent WHO standards for improving the care of small and sick newborns in health facilities, and WHO recommendations for care of the preterm and low-birthweight infant, have collated evidence to support clinical interventions for SSNC [2, 3]. This package of care, known as SSNC level-2 + , includes ten clinical interventions, such as thermal care provision, safe administration of oxygen, and respiratory support with continuous positive airway pressure (CPAP). This care is potentially high impact, saving around 750,000 lives per year, but requires major health systems scale-up, especially in high-burden settings [1, 2].

To speed up progress, *Every Newborn* Action Plan (ENAP) together with Ending Preventable Maternal Mortality (EPMM) set maternal and newborn coverage targets for 2020–2025, including for antenatal visits, skilled birth attendants, postnatal care, and SSNC at national and sub-national levels [4]. The fourth ENAP coverage target for SSNC is for 80% of districts in every country to have at least one functional level-2 inpatient newborn care unit with CPAP by 2025 [4].

To assess progress towards this ENAP coverage target for level-2 + newborn units, countries need methods to measure hospital readiness, and benchmark between facilities and countries and over time. Health facility service readiness tools are widely used, for example to measure basic and comprehensive emergency obstetric care and wider service provision [5–7]. These service readiness tools do not comprehensively assess level-2 + SSNC. A health facility assessment (HFA) tool for SSNC, co-designed by the NEST360 alliance and United Nations Children’s Fund (UNICEF) in partnership with four African governments, can assess readiness to provide SSNC [8].

There are a range of approaches to score service readiness data [9]. However, there are no standard methods to quantitatively summarise level-2 + SSNC service readiness and measure progress towards these global newborn coverage targets. Some content specific tools do give standard scoring or indexing methods, such as for the WHO infection prevention and control assessment [10]. However, these are content-specific and not applicable to level-2 + SSNC. Programme implementation can also be assessed by scoring, but there are a wide range of scoring approaches and no consensus on the ideal approach [11]. Patient-level clinical scoring systems are widely used to predict individual level outcomes, but do not apply to service readiness of a given hospital and linked health system [12]. Tracer indicators are also used, including for the WHO Service Availability and Readiness Assessment (SARA), which does not include indicators for more comprehensive newborn care [6]. Signal functions are commonly applied to measure basic and comprehensive emergency obstetric care [5, 13]. However, there is as yet no evidence base to inform signal functions for level-2 + SSNC. Ongoing revisions to the emergency obstetric care (EmOC) monitoring framework aim to include expert-informed newborn signal functions for level-2 + care [14, 15]. To fill this gap in service readiness measurement for level-2 + SSNC, two new scoring approaches were developed.

This paper is part of a supplement reporting findings and learnings from Newborn Essential Solutions and Technologies (NEST360), an alliance of partners, including four African governments (Kenya, Malawi, Nigeria, and Tanzania), working to reduce neonatal inpatient deaths by improving level-2+ newborn care in hospitals. The series provides reviews of tool design, analyses, and learning for implementing level-2 + SSNC.

### Aim

In this paper, we report the development and application of two scoring approaches to assess hospital service readiness for SSNC using a large HFA dataset from 68 neonatal units in 64 hospitals in Kenya, Malawi, Nigeria, and Tanzania. Specifically, we cover the following three objectives:

- Objective 1: Standards-based scoring: develop and evaluate standards-based service readiness for SSNC, and identify strengths and gaps by adapted WHO Health System Building Blocks (HSBBs).
- Objective 2: Level-2 + scoring: develop and evaluate service readiness for WHO level-2 + and transition clinical interventions to inform tracking of ENAP coverage target four.
- Objective 3: Comparison: To evaluate the consistency of standards-based and level-2 + scores by comparing the two scoring approaches by facility and country.

## Methods

### NEST360 alliance

NEST360, an alliance of partners, including four African governments (Kenya, Malawi, Nigeria, and Tanzania), established in 2019, aims to reduce newborn deaths in hospitals by adopting a co-created health systems package with innovative technologies, mentoring for clinicians and engineers, and evidence-based implementation strategies for sustainability. The health systems package has been implemented in 68 neonatal units with 13 in Kenya, 37 in Malawi, 11 in Nigeria (at 7 hospitals), and 7 in Tanzania. 

### Data source and data collection

The HFA tool for SSNC, co-designed by NEST360 and UNICEF in partnership with four African governments, was systematically developed using a three-step evidence-based process to review existing standards and establish a list of health systems ingredients for SSNC, scope existing service readiness tools, and co-design, refine, and operationalise a new HFA tool for level-2 + SSNC [8]. The tool includes ten discrete modules which are aligned with the adapted HSBBs, including the following areas: 1) facility infrastructure; 2) neonatal unit infrastructure; 3) pharmacy and laboratory; 4) medical devices and supplies; 5) biomedical technician workshop; 6) human resources; 7) information systems; 8) leadership and governance; 9) family-centred care; and 10) hand hygiene observation. There are 1508 items overall across six adapted HSBBs. The tool was used to collect data on hospital readiness pre-NEST360 implementation at 68 neonatal units in 64 hospitals implementing with NEST360 in Kenya, Malawi, Nigeria, and Tanzania during the period September 2019—March 2021. HFA data collection was completed in one day at each hospital using a mobile REDCap application on Android tablets [16]. Data collectors were trained for four days before data collection began. Data were verified by HFA team supervisors and the NEST360 country database manager. All HFA data were synced to and stored on servers of the designated country partner during and after data collection. De-identified HFA data were transferred to a central database for analysis.

### Methods by objectives

#### Objective 1: Standards-based scoring: develop and evaluate standards-based service readiness for SSNC, and identify strengths and gaps by adapted WHO HSBBs

##### Development of framework for objective 1

The framework for this approach was developed based on the HSBB framework adapted from WHO with a focus on six HSBBs [17]. The six adapted HSBB modules focus on: 1) infrastructure; 2) medical devices and supplies; 3) human resources; 4) information systems; 5) family-centred care; and 6) governance. Sub-modules for each HSBB were also identified, and are further described in Table 1. Service readiness was assessed according to national and global standards for SSNC. All items required for standards-based service readiness of SSNC, where a clear national or global standard exists, were included in the analysis. Items that did not have a clear national or global standard were excluded from analyses. Items were grouped into HSBB modules and sub-modules to support identification of health systems gaps for action (Table 1, Additional File 1).

**Table 1:** Health system building block (HSBB) modules and sub-modules: description of categories included in standards-based scoring

HSBB and components	Description
**1. Medical devices and supplies (544/630 items included)**	
Medical device requirements	• Device availability and functionality • Consumable availability and stockouts • Infection prevention supply availability and stockouts
Laboratory	• Laboratory infrastructure • Equipment availability and functionality • Laboratory supply availability and stockouts • Laboratory test and guideline availability • Laboratory staffing
Pharmacy	• Medicine availability and stockouts • Supply chain processes
Biomedical workshop	• Repair tool availability and functionality • Spare part availability and stockouts • Workshop infrastructure • Planning and management processes • Preventive maintenance processes
**2. Human resources (229/381 items included)**	
People	• Staffing allocation and staff numbers
Education	• Clinical and technical staff training • Provision of clinical competencies
Enabling Environment	• Guideline availability and accessibility • Support and supervision • Hospital policies and working conditions • Provision of free newborn care services
**3. Infrastructure (131/221 items included)**	
Electrical power	• Backup power sources and functionality • Neonatal unit power infrastructure • Electricity availability by hospital area
Medical gases and vacuum	• Hospital oxygen systems • Neonatal unit walled and piped oxygen availability
Referral	• Communication method availability and functionality • Transport method availability and functionality • Transport maintenance and support systems
Space and design	• Neonatal unit capacity • Dedicated areas in neonatal unit • Temperature and heating in neonatal unit • Fire prevention in neonatal unit • Staff/visitor personal items and dedicated areas • Biomedical workshop space availability
Water, Sanitation, and Hygiene	• Hospital water infrastructure • Water availability by hospital area • Autoclaving availability and functionality • Hand hygiene options on the neonatal unit • Toilet/latrine options for neonatal unit staff/visitors • Neonatal unit sterilisation and equipment disinfection • Waste management on the neonatal unit • Infection surveillance • Observed hand hygiene behaviour
**4. Information Systems (95/215 items included)**	
Data collection	• Forms/registers used on the neonatal unit • Register completion • Summary reports used • Maternal perinatal death surveillance and response
Data management	• Filing systems on the neonatal unit • Indicators used at the hospital • Summary data for reporting • Civil registration and vital statistics
Maternal and perinatal death surveillance and response (MPDSR)	• MPDSR reporting
Foundations	• Form/register supply and stockouts • Filing systems on the neonatal unit • Electronic information system availability and management • Infrastructure for electronic information systems
**5. Family-Centred Care (30/40 items included)**	
Organisation of care	• Guideline availability and accessibility • Sitting, sleeping, and visitor infrastructure • Dedicated areas for mothers/families
Discharge and early development	• Guideline availability and accessibility
Parent power	• Guideline availability and accessibility
Kangaroo mother care (KMC)	• KMC infrastructure and occupancy
**6. Governance (14/21 items included)**	
Hospital management	• Clinical audit and management meetings • Staff absenteeism and training plans • Hospital and neonatal unit target setting • Financial management policies

##### Data analyses for objective 1

HFA data were cleaned, and quality checked in REDCap and during analysis using Stata 17 (StataCorp LLC, Texas, USA). Items were scored on availability (e.g., nurses staffed on the neonatal unit), or if relevant on availability and functionality (e.g., devices). Functionality was defined according to the item. For example, functional devices must be in working order, and functional clinical guidelines must be available and easily accessible at the time of the HFA. No points were given if not available, one point if available, and two points if available and functional. Scores were calculated by HSBB with a percentage based on total score over the total possible score for that HSBB. Items with missing responses were removed from the denominator to be conservative. A sensitivity analysis was performed to determine the impact of removing items with missing responses from scores compared to assigning missing items a score and keeping them in the denominator. Scores were analysed using the neonatal unit as the unit of analysis. At the country level, scores were aggregated using the median and interquartile range of all neonatal unit scores in that country.

The six HSBB modules (i.e., infrastructure, medical devices and supplies, human resources, information systems, family-centred care, and governance) contributed equally to the neonatal unit total percentage score and were summarised using heatmaps and presented by country, HSBB module and sub-modules. Heatmaps present scores using five colours to represent each 20% increase in scores, with 0–20% as lowest (dark red) and 80–100% as highest (green).

#### Objective 2: Level-2 + scoring: develop and evaluate service readiness for WHO level-2 and transition clinical interventions to inform tracking of ENAP coverage target 4

##### Development of framework for objective 2

Service readiness criteria for assessment of WHO level-2 SSNC and transition to level-3 care were developed through selection of ten interventions according to the WHO levels of care framework for level-2 + SSNC (Additional File 2) [1]. Level-2 + SSNC includes seven level-2 interventions: 1) thermal care; 2) intravenous and assisted feeding; 3) safe oxygen use; 4) provision of antibiotics guided by culture report; 5) jaundice management with phototherapy; 6) management of neonatal encephalopathy; and 7) referral for congenital abnormalities. Level-2 + SSNC also includes three transition to level-3 interventions: 1) CPAP use; 2) exchange transfusion; and 3) follow-up of at-risk newborns. This focus on level-2 + SSNC is aligned with the content of the NEST360/UNICEF HFA tool and the ENAP coverage target for 80% of districts in every country to have at least one functional level-2 + inpatient newborn care unit with respiratory support by 2025, which requires evaluation of level-2 + care to assess progress [4, 18].

For each clinical intervention, all items needed for provision of care and other health systems resources were identified. First, NEST360 clinical education modules and other national and global clinical guidelines and educational materials were used to understand the processes for each clinical intervention, including the use of medications, devices, consumables, and any required health systems resources, such as specialist staff training or required infrastructure [2, 19–22]. Second, items needed for each intervention were reviewed in consultation with two medical doctors (EMM, JEL) and two nurses (EG, DG) with specialist training and experience in SSNC in NEST360 implementing countries. Sub-modules required for diagnosis/screening and treatment/management were identified (Table 2, Additional File 3). This included any items needed to assess the baby before intervention, and any individual pathways to providing care (e.g. cup feeding, NG tube feeding). For each sub-module, individual items needed for that pathway were identified.

**Table 2:** Clinical interventions, and diagnosis and treatment sub-modules included in WHO level-2+ small and sick newborn care scoring

	**Clinical Intervention**	**Diagnosis and treatment/management sub-modules**
WHO Level-2 small and sick newborn care	1. Thermal care including KMC for all stable neonates < 2000 g (59 items included)	*Diagnosis* • Temperature monitoring for baby *Treatment/management* • Items for thermal support • Kangaroo Mother Care (KMC) • Devices for thermal support • Device power sources • Infection prevention • Infrastructure for thermal support • Guidelines, initiation of care, and training
	2. Assisted feeding and intravenous (IV) fluids (107 items included)	*Diagnosis* • Blood glucose screening *Treatment/management* • Breast feeding and milk banking • Cup feeding • Nasogastric (NG) tube feeding • IV fluids • IV fluids equipment and consumables • Device power sources • Infection prevention • Guidelines, initiation of care, and training
	3. Safe administration of oxygen (64 items included)	*Diagnosis* • Oxygen assessment • Vital sign monitoring *Treatment/management* • Items for oxygen provision • Oxygen sources, including devices • Device power sources • Infection prevention • Guidelines, initiation of care, and training
	4. Detection and management of neonatal sepsis with injection antibiotics (42 items included)	*Diagnosis* • Readiness for culture • Temperature monitoring for baby *Treatment/management* • Antibiotics • Guidelines, initiation of care, and training
	5. Detection and management of neonatal jaundice with phototherapy (69 items included)	*Diagnosis* • Bilirubin measurement • Laboratory can assess underlying causes • Other monitoring for baby *Treatment/management* • Equipment for phototherapy provision • Consumables for phototherapy provision • Device power sources • Therapeutic irradiance • Infection prevention • Guidelines, initiation of care, and training
	6. Detection and management of neonatal encephalopathy (29 items included)	*Diagnosis* • Diagnostics *Treatment/management* • Seizure management • Therapeutic hypothermia • Guidelines, initiation of care, and training
	7. Detection and referral/management of congenital abnormalities (23 items included)	*Treatment/management* • Referral communication systems • Referral transport systems • Guidelines, initiation of care, and training
Transition from WHO level-2 to level-3 small and sick newborn care	8. CPAP management of preterm respiratory distress (80 items included)	*Diagnosis* • Oxygen assessment • Vital sign monitoring *Treatment/management* • Items for CPAP • Equipment for CPAP • Oxygen sources, including devices • Device power sources • Infection prevention • Guidelines, initiation of care, and training
	9. Perform exchange transfusion for a newborn (63 items included)	*Diagnosis* • Bilirubin measurement • Laboratory can assess underlying causes • Other monitoring for baby *Treatment/management* • Equipment for exchange transfusion • Blood bank support for transfusion • Infection prevention • Guidelines, initiation of care, and training
	10. Provide follow-up of at-risk newborns (3 items included)	*Treatment/management* • Guidelines and discharge plan

*Abbreviation:* *CPAP *Continuous Positive Airway Pressure

##### Data analysis for objective 2

Sub-modules for each clinical intervention were scored on a zero to three scale – allocating zero points if "not ready", one point if "basic readiness", two points if "comprehensive readiness", and three points for "gold standard care". Basic, comprehensive, and gold-standard readiness were defined according to national and global clinical guidelines, NEST360 educational materials, and review by clinicians with SSNC experience as described above. These sub-module scores included individual items needed for each individual pathway, however, scores were allocated for the overall sub-module. For sub-modules with missing responses, missing items were removed from the analysis to be conservative and allow all neonatal units to achieve the highest possible score regardless of missing data. Sub-module scores were added to calculate an intervention-level score. Intervention scores were calculated by a percentage based on total sub-module score over the total possible sub-module score for that intervention. Follow-up of at-risk newborns, which only included clinical guidelines, was excluded from the overall score to be conservative. Therefore, nine clinical interventions (thermal care, intravenous and assisted feeding, safe oxygen use, provision of antibiotics guided by culture report, jaundice management with phototherapy, management of neonatal encephalopathy, referral for congenital abnormalities, CPAP use, and exchange transfusion) were set to contribute equally to the neonatal unit total percentage score and were summarised using heatmaps and presented by country and clinical intervention. Heatmaps present scores using five colours to represent each 20% increase in scores, with 0–20% as lowest (dark red) and 80–100% as highest (green). Stacked bar charts present scores demonstrating the number of sub-modules with no readiness (dark red), basic readiness (orange), comprehensive readiness (yellow), and gold-standard readiness (green). Two additional categories were assessed to evaluate other clinical care items and health systems resources needed for the clinical interventions, but these were not included in the overall level-2 + scores, which focused on interventions only. Scores were analysed using the neonatal unit as the unit of analysis. At the country level, scores were aggregated using the median and interquartile range of all neonatal unit scores in that country.

#### Objective 3: Comparison: To evaluate the consistency of standards-based and level-2 + scores by comparing the two scoring approaches by facility and country

A comparison of standards-based and level-2 + scores were evaluated visually using a scatter plot to assess consistency by facility and country, and a boxplot to show differences in median scores and score ranges. Comparison between the two scores was also formally assessed using correlation analysis.

### Ethical approval

Ethical approval was received in each country from a local institutional review board (Additional File 4) and the London School of Hygiene and Tropical Medicine ethics committee (no. 21892). The NEST360 alliance data sharing agreement covered data sharing between organisations. No individual consent was required for the study as no personal identification data were included.

## Results

Data from 68 neonatal units at 64 hospitals were analysed, including 13 in Kenya, 37 in Malawi, 11 in Nigeria (at 7 hospitals), and 7 in Tanzania. Thirty-four were neonatal units at secondary and tertiary hospitals and 34 were units at primary hospitals, though most primary hospitals included were in Malawi (94%). 

### Results by objectives

#### Objective 1: Standards-based scoring: develop and evaluate standards-based service readiness for SSNC, and identify strengths and gaps by adapted WHO HSBBs

**Fig. 1:**
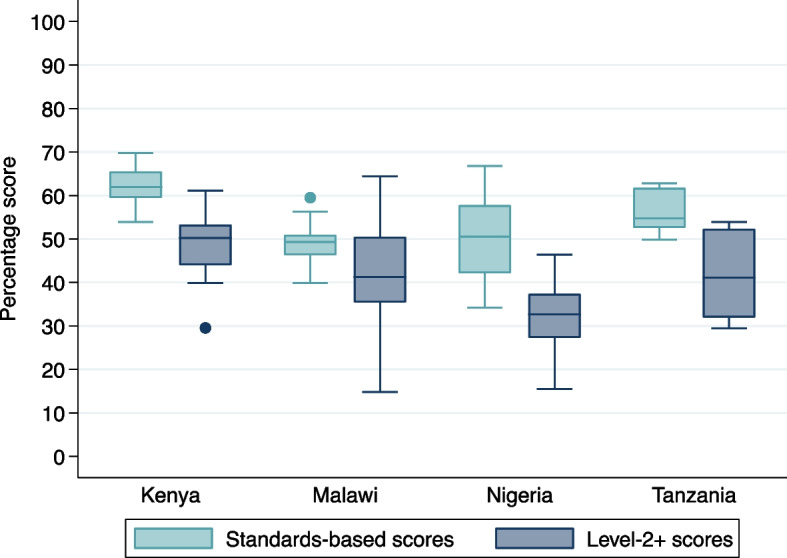
Boxplot of standards-based and WHO level-2+ service readiness scores overall by country for 68 neonatal units at 64 hospitals implementing with NEST360 at baseline. Abbreviations: WHO - World Health Organization

Of 1508 items included in the HFA tool, 1043 (69%) were included in standards-based scoring. This includes all items required for standards-based service readiness of SSNC, where a clear national or global standard exists. The overall median score was 51% [IQR 48–57%], with some variation by country: 62% [IQR 59-66%] in Kenya, 49% [IQR 46-51%] in Malawi, 50% [IQR 42-58%] in Nigeria, and 55% [IQR 53-62%] in Tanzania (Fig. 1). Of the 68 neonatal units, 27 had overall scores of < 50% and 41 had scores of 50–75%. No neonatal units achieved scores > 75%. Individual HSBB scores varied from 0% for family-centred care in Malawi to 100% for governance in Kenya.

**Fig. 2:**
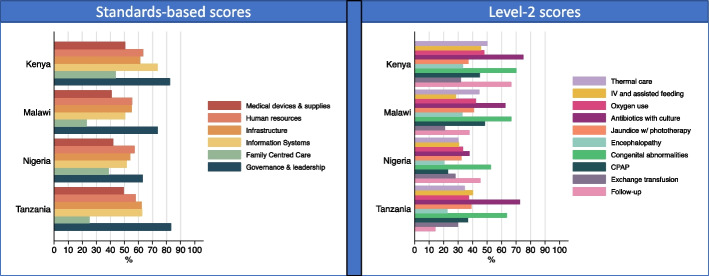
Percent readiness by health system building blocks and level-2 + clinical interventions at baseline. Abbreviations: IV – Intravenous; CPAP—Continuous Positive Airway Pressure

**Fig. 3:**
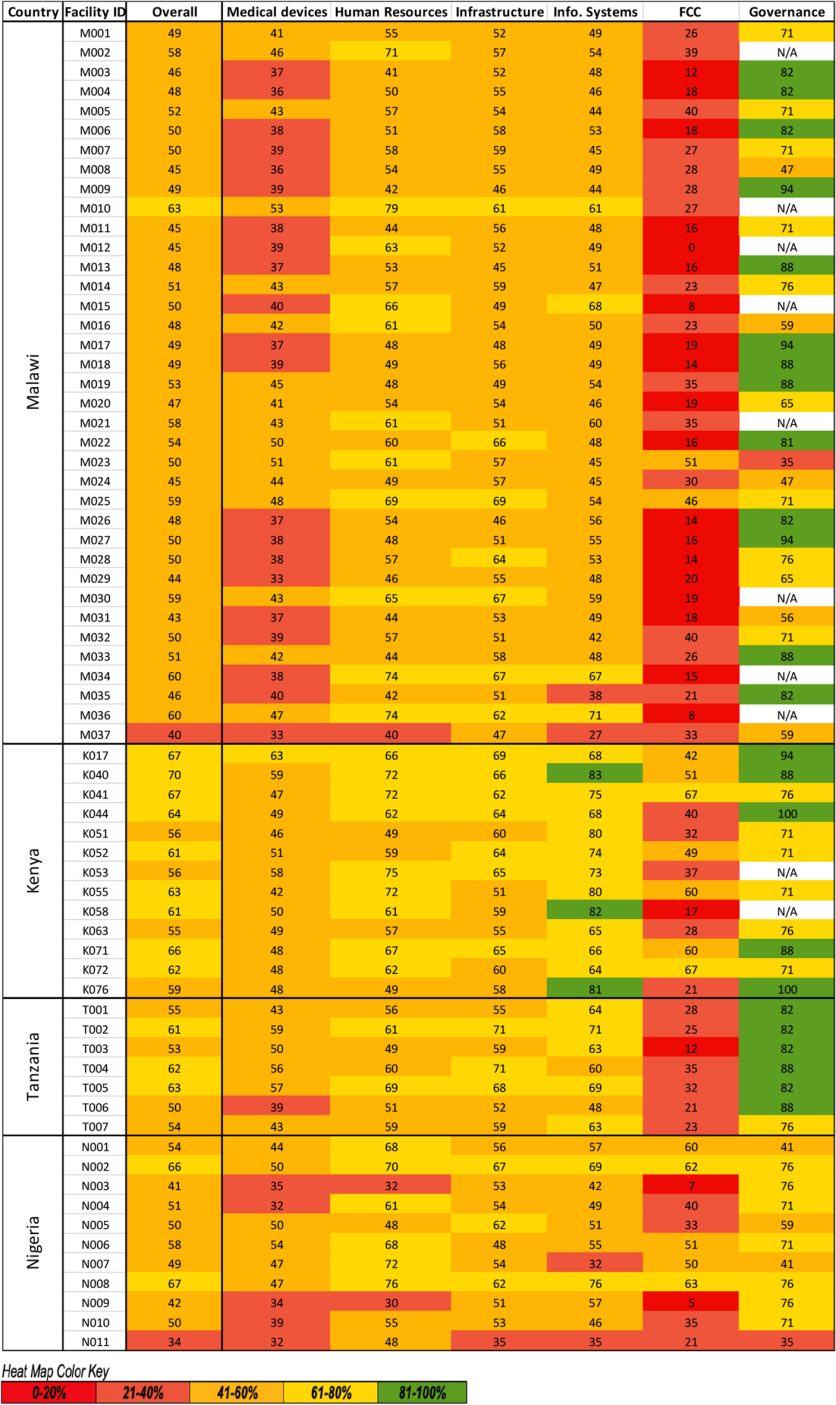
Heat map of standards-based service readiness scores overall and by health system building block for 68 neonatal units at 64 hospitals implementing with NEST360 at baseline. Abbreviations: Info. Systems – Information Systems; FCC – Family-Centred Care; N/A - Not applicable

Across all countries, family-centred care was the lowest scoring HSBB [27%, IQR 18–40%] while governance was the highest scoring HSBB [76%, IQR 71–82%] (Fig. 2). Medical device scores were also low across most neonatal units with 53 (78%) neonatal units having scores of < 50% [43%, IQR 38–48%], though scores were somewhat higher in Kenya [49%, IQR 48–51%] and Tanzania [50%, IQR 43–57%]. Neonatal units in Kenya had higher scores for information systems [74%, IQR 68–80%] than neonatal units in other countries [54%, IQR 48–66%] (Fig. 3). Neonatal unit-level heat maps revealed gaps in specific HSBB sub-modules by highlighting which areas had low scores of less than 20% (dark red) and 20–40% (light red), particularly the biomedical workshop, staff allocation and numbers, and family-centred care policies and infrastructure (Additional File 5).

The sensitivity analysis performed to determine the impact of removing items with missing responses from scores showed a small increase from 41% [IQR 34–48%] to 56% [IQR 48–61%] in the human resources HSBB scores when missing values were removed from the analysis for a subset of 58 neonatal units. There were no differences for medical device, family-centred care, and governance scores, and no notable differences for infrastructure [Pre: Median 55%, IQR 51–61%; Post: Median 56%, IQR 52–62%] or information systems scores [Pre: Median 51%, IQR 47–63%; Post: Median 51%, IQR 47–64%].

#### Objective 2: Level-2 + scoring: develop and evaluate service readiness for WHO level-2 and transition clinical interventions to inform tracking of ENAP coverage target 4

Of 1508 items included in the HFA tool, 309 (20%) were included in level-2 and transition care scoring. Overall median score was 41% [IQR 35–51%] of items for clinical interventions, with 50% [IQR 44–53%] in Kenya, 41% [IQR 35–50%] in Malawi, 33% [IQR 27–37%] in Nigeria, and 41% [IQR 32–52%] in Tanzania (Fig. 1). Of the 68 neonatal units, 48 had overall scores of < 50%, and 20 had scores of 50–75%. No neonatal units achieved high scores of > 75%. Individual intervention scores varied from 0% for management of neonatal encephalopathy in Nigeria to 100% for provision of antibiotics guided by culture report in Malawi and Kenya. Overall, hospitals were not ready or only had a basic readiness to provide level-2 + care (Additional File 6).

**Fig. 4:**
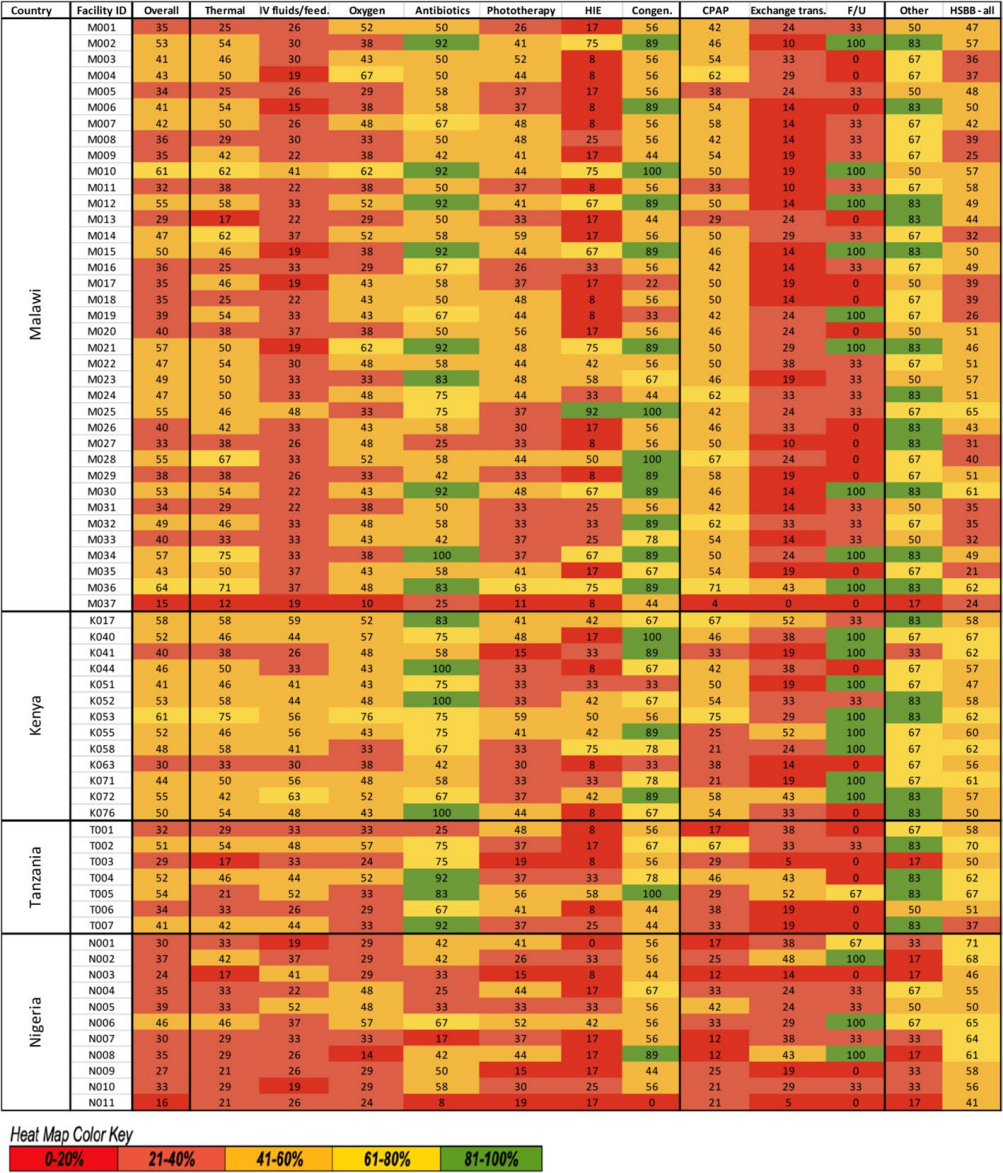
Heat map of level-2 + service readiness scores overall and by intervention for 68 neonatal units at 64 hospitals implementing with NEST360 at baseline. Abbreviations: Thermal – Thermal care; IV fluids/feed.—intravenous and assisted feeding; Oxygen – safe oxygen use; Antibiotics—provision of antibiotics guided by culture report; Phototherapy—jaundice management with phototherapy; HIE—management of neonatal encephalopathy; Congen.—referral for congenital abnormalities; CPAP—Continuous Positive Airway Pressure; Exchange trans. – Exchange transfusion; F/U – follow-up of at-risk newborns; HSBB – health system building blocks

Overall, readiness to provide antibiotics guided by culture report was the highest scoring intervention [58%, IQR 50–75%]; however, this was only the highest scoring intervention in neonatal units in Kenya and Tanzania (Fig. 4). In contrast, detection and management of neonatal encephalopathy was the lowest scoring intervention and had the most variation across neonatal units compared to the other interventions [21%, IQR 8–42%, SD 23%]. Readiness to provide intravenous fluids and assisted feeding was also low [33%, IQR 26–41%], though these scores were higher in neonatal units in Kenya [44%, IQR 41–56%] and Tanzania [44%, IQR 33–48%]. Readiness to perform exchange transfusion, an intervention in the transition from level-2 to level-3 care, was low [24%, IQR 14–33%] compared to other interventions, and this was consistent across countries. Readiness to provide CPAP was higher in Malawi [50%, IQR 42–54%] compared to other countries [Kenya 46%, IQR 33–54%; Nigeria 21%, IQR 12–33%; Tanzania 33%, IQR 29–46%].

### Objective 3: Comparison: To evaluate the consistency of standards-based and level-2 + scores by comparing the two scoring approaches by facility and country

**Fig. 5:**
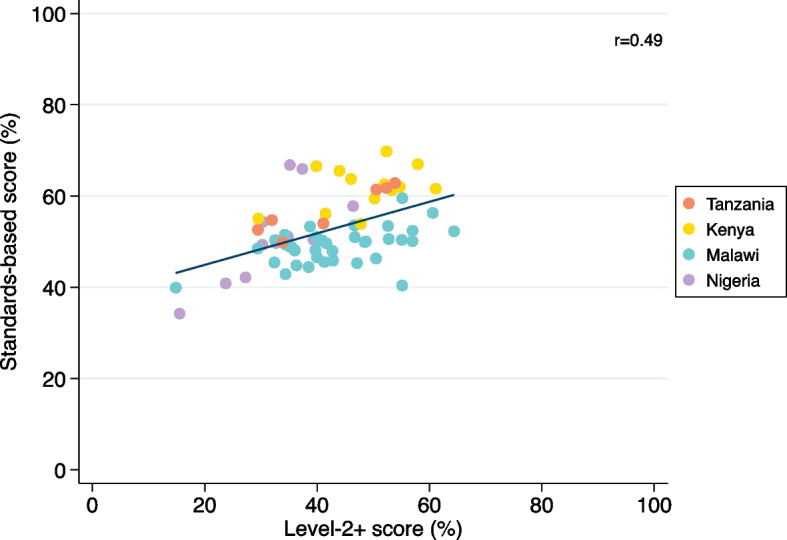
Scatterplot of standards-based and WHO level-2+ service readiness scores overall by country for 68 neonatal units at 64 hospitals implementing with NEST360 at baseline. Abbreviations: WHO - World Health Organization

Level-2 + scores included fewer items than standards-based scores. Standards-based scores were higher than level-2 + scores in all neonatal units in Kenya, Nigeria, and Tanzania (Fig. 5). Standards-based scores were lower in nine neonatal units (24%) in Malawi. Neonatal units had a median difference of 11% [IQR 5–17%] in overall scores, with 11% [IQR 8–18%] in Kenya, 7% [IQR 1–13%] in Malawi, 17% [IQR 15–24%] in Nigeria, and 13% [IQR 9–23%] in Tanzania.

Hospitals in Malawi had wider dispersion of level-2 + scores [Range 15–64%] compared to standards-based scores [Range 40–59%]. Median sub-module scores in all countries had wider dispersion of standards-based scores [Range 27–76%] compared to level-2 + scores [Range 20–58%]. Standards-based and level-2 + scores were moderately correlated overall [*r* = 0.49]. Scores were strongly correlated in Tanzania [*r* = 0.92] and Nigeria [*r* = 0.76], and less correlated in Malawi [*r* = 0.49] and Kenya [*r* = 0.38].

## Discussion

We developed two summary scoring approaches: a) standards-based, including items for SSNC service readiness by HSBB, and scored on availability and functionality, and b) level-2 +, scoring items on readiness to provide WHO level-2 + clinical interventions. For 68 neonatal units in Kenya, Malawi, Nigeria, and Tanzania, both these scoring approaches revealed major gaps in readiness to provide level-2 + SSNC, which is required to reach a neonatal mortality rate of less than 12 per 1000 live births and meet Sustainable Development Goal 3.2. Standards-based scoring, counting items or ingredients, revealed low scores overall with neonatal units having only half of items required for care [51%, IQR 48–57%], and with most neonatal units [*n* = 41, 60%] having intermediate overall readiness scores of 50–75%. Level-2 + scoring, based on items for nine level-2 + clinical interventions, revealed even lower scores with neonatal units having roughly 40% of items required for clinical interventions [41%, IQR 34–52%], and with most neonatal units [*n* = 48, 71%] having low overall readiness scores of < 50%. Though the two scoring approaches can not be directly compared as they provide different measures of SSNC service readiness, standards-based and level-2 + scores were consistent and moderately correlated overall (*r* = 0.49). However, there was wider dispersion of level-2 + intervention scores [Range 15–64%].

Our study found substantial gaps in readiness to provide SSNC, which is aligned with findings from similar studies noting major gaps in readiness to provide newborn care, though comparison of our scoring approach to other measurement approaches for service readiness for SSNC is challenging given differences in methodology [13, 23, 24]. One study assessed readiness to provide maternal and newborn care signal functions in facilities in Nigeria, Ethiopia, and India, noting that only a third of Nigerian facilities in 2012, and a fifth of Nigerian facilities in 2015, were ready to provide clean cord care for newborns (a signal function) [13]. Clean cord care is not part of level-2 + newborn care, but the findings of low readiness for newborn care are similar to our findings. Another study from Kenya assessing readiness to provide newborn care in the labour and delivery units and newborn units at facilities found results that differ. They found that all 31 facilities in Nairobi City County had more than 50% of required items for care; however, this study primarily assessed readiness to provide newborn care on the labour and delivery unit, and only assessed neonatal unit equipment and drugs, which had lower scores, and was more consistent with our results [23]. Another study assessing availability of staffing, equipment, consumables, and infrastructure for newborn care at facilities in South Africa noted that many facilities were missing key items at baseline, and noted ongoing challenges with availability of appropriate infrastructure, staffing, and equipment [24].

Standards-based and level-2 + scoring approaches can both be useful to identify actionable gaps to improve readiness to provide interventions. For example, medical device scores at baseline, pre-NEST360 intervention, were low across most hospitals, particularly with device availability and functionality, and readiness to provide preventive or corrective maintenance. A focus of the NEST360 alliance is to improve newborn medical device and maintenance readiness in hospitals. Lack of appropriate device maintenance can lead to hospitals having large numbers of non-functional equipment known as “equipment graveyards”, limiting the ability to provide life-saving interventions that require medical devices [25, 26]. Level-2+ scores are also useful for understanding gaps in readiness. Thermal care is an important intervention for many small and sick babies. Improvements in practices such as kangaroo mother care (KMC), including KMC initiated immediately after birth (iKMC), and in thermal care infrastructure could have measurable impacts on newborn outcomes [3, 27, 28]. On average, neonatal units had fewer than half of items needed for thermal care infrastructure and fewer than one third of items needed to provide KMC to newborns. Thermal care scores highlighted that many hospitals are able to appropriately assess and monitor newborn temperatures, but lack the appropriate infrastructure to support thermal care, especially the power and backup power infrastructure required to operate warming devices (e.g., radiant warmers, incubators, heated cots). In addition, readiness to provide KMC was low with many neonatal units lacking adequate infrastructure and support, and sufficient space.

Summarising and scoring service readiness data is important to assess progress towards global targets for newborn care, including the fourth ENAP coverage target for 80% of districts to have at least one level-2 + newborn care unit by 2025 [4]. In addition, scores are important for hospitals and programmes to identify actionable gaps in readiness to provide care. There are currently no standard approaches for scoring health facility service readiness data. These analyses demonstrate the findings from two methods to quantitatively summarise a large service readiness dataset for SSNC.

### Implications for hospitals and programmes

Family-centred care was the lowest-scoring HSBB in standards-based scoring. Implementing family-centred care in hospitals can reduces stress on families and improve newborn outcomes. In standards-based scoring, major gaps in readiness to provide family-centred care could highlight opportunities for focused interventions. For example, many hospitals lacked adequate infrastructure and policies to provide family-centred care, including space and appropriate furniture for KMC, and had restrictive family visiting policies. WHO recommends KMC for all stable low-birthweight babies, and there is strong evidence suggesting that this improves newborn outcomes [3, 29]. In addition, WHO recommends that parents have unrestricted access to their newborns to support feeding and bonding, which are both important after discharge [2, 3]. Governance scores were high overall, though it is worth noting that governance scores primarily assessed hospital policies, and availability and frequency of hospital management team meetings, such as for quality improvement teams, rather than implementation of policies.

Newborns are especially vulnerable to healthcare-associated infections, the majority of which are antimicrobial resistant and associated with increased mortality risk. In level-2 + scoring, readiness to provide antibiotics guided by sample culture report was a major strength across hospitals. This was largely driven by availability of appropriate antibiotics with no stockouts and temperature monitoring for infection. These results confirm narratives that hospitals can provide culture results, though their use to guide clinical management remains underutilised [30]. Hospitals may document few culture results though many have the necessary items to do them [31]. The level-2 + score results could support hospitals and programmes in identifying how to increase the clinical use of culture.

### Strengths and limitations

One strength of this paper is that the two scoring methods were applied to a large dataset with HFA data from 68 neonatal units in 64 hospitals in four countries. The scores concisely summarises a large dataset, rather than only including a few tracer indicators. This is the first study to our knowledge that has systematically developed scoring approaches for evaluating service readiness data for SSNC, linked to existing global frameworks and guidelines, including an adapted HSBB framework, the WHO standards for improving the care of small and sick newborns in health facilities, and WHO recommendations for care of the preterm and low-birthweight infant [2, 3, 17]. Results and visualisations developed from these scoring methods can be used by hospitals and programmes to identify health systems gaps, and by governments to inform tracking of national and global newborn care targets, such as the ENAP coverage targets.

One limitation is that for standards-based scores, not all HFA items were included, particularly where there is no clear standard (e.g., staff to baby ratios). For level-2 + scores, it is challenging to assess readiness to provide some interventions through standard HFAs, particularly interventions like detection and management of congenital abnormalities, which can vary widely depending on the abnormality. For both scoring methods, it is unknown if these service readiness scores are associated with SSNC quality measures and patient-level outcomes, such as mortality.

### Future research

These summary scoring approaches could be useful for further analyses to understand the associations between service readiness and patient-level outcomes. Analyses could evaluate these service readiness inputs against patient-level outcomes, such as mortality or coverage of an important clinical intervention such as CPAP, to provide more robust evidence if these service readiness inputs predict outcomes. Scores that have more association with outcomes may be more useful and actionable. They may also require fewer items and be more sensitive to health systems inputs. This could allow targeted opportunities to improve readiness for the most important level-2 + interventions, and for learning from high-performing neonatal units. Given limited resources in many settings, identifying opportunities to allocate health systems resources to high-impact interventions could lead to greater change in mortality.

Future analyses could explore validation of the two scoring approaches, and also determine which items are most directly linked with quality of small and sick newborn care to help identify a smaller set of items that are most important for routinely tracking health systems gaps.

## Conclusion

Standards-based and level-2 + scoring approaches could be used to assess progress towards global targets for SSNC, including the ENAP coverage target. Scores based on readiness to provide SSNC level-2 + clinical interventions were lower and with a wider range, and may be more distinguishing. Importantly, this scoring also involved fewer variables and hence the HFA tool could be further reduced. Wider use of this tool and quantification would be more robust than self-report of how many countries meet the ENAP coverage target.

Neonatal deaths represent almost half of under-five child deaths, and improved newborn survival with good long term outcomes is important to achieve global targets, including the fourth ENAP coverage target for SSNC [4]. Many countries are off-track to reach this coverage target and Sustainable Development Goal 3.2. To save lives, these data and scores are useful for informing governments on where gaps exist and potential areas of focus in the health system for programmes aimed at improving SSNC. It is imperative that countries are supported to assess actionable gaps for change and objectively measure progress, accelerating their progress to reduce neonatal mortality.

## Supplementary Information

**Additional file 1.** Items included in standards-based service readiness score by health system building blocks.

**Additional file 2.** Items included in standards-based service readiness score by health system building blocks.

**Additional file 3.** Items included in level-2+ service readiness score by clinical intervention.

**Additional file 4.** Local ethical approval for the complex evaluation of the implementation of a small and sick newborn care package with NEST360.

**Additional file 5.** Heat map of standards-based service readiness scores by HSBB module and sub-modules for 68 neonatal units at 64 hospitals implementing with NEST360 at baseline.

**Additional file 6.** Stacked bar chart of level-2+ service readiness scoring sub-modules demonstrating overall readiness to provide level-2+ clinical interventions.

## Data Availability

Data sharing and transfer agreements were jointly developed and signed by all collaborating partners in the NEST360 alliance. The dataset generated during the current study will be available upon request subject to approval by the NEST360 learning network and collaborating parties. The HFA tool, data dictionary, and associated materials are available from the NEST360/UNICEF Implementation Toolkit for Small and Sick Newborn Care (https://www.newborntoolkit.org) and NEST360 website (https://nest360.org).

## Abbreviations

CPAP: Contiuous Positive Airway Pressure
EmONC: Emergency Obstetric and Newborn Care
ENAP: Every Newborn Action Plan
EPMM: Ending Preventable Maternal Mortality
HFA: Health Facility Assessment
HSBB: Health System Building Block
IV: Intravenous
KMC: Kangaroo mother care
LSHTM: London School of Hygiene & Tropical Medicine
MoH: Ministry of Health
NEST360: Newborn Essential Solutions and Technologies
SARA: Service Availability and Readiness Assessment
SSNC: Small and Sick Newborn Care
UNICEF: United Nations Children's Fund
WHO: World Health Organization
