# Supplementary material for: Quantifying health facility service readiness for small and sick newborn care: comparing standards-based and WHO level-2 + scoring for 64 hospitals implementing with NEST360 in Kenya, Malawi, Nigeria, and Tanzania
Source: BMC Pediatr. 2024 Mar 12;23(Suppl 2):656. doi: 10.1186/s12887-024-04578-5 (PMC10935770; doi:10.1186/s12887-024-04578-5)
Supplement: Supplementary file 2 — Additional file 2. Items included in standards-based service readiness score by health system building blocks. [file 12887_2024_4578_MOESM2_ESM.pptx]

## Slide 1
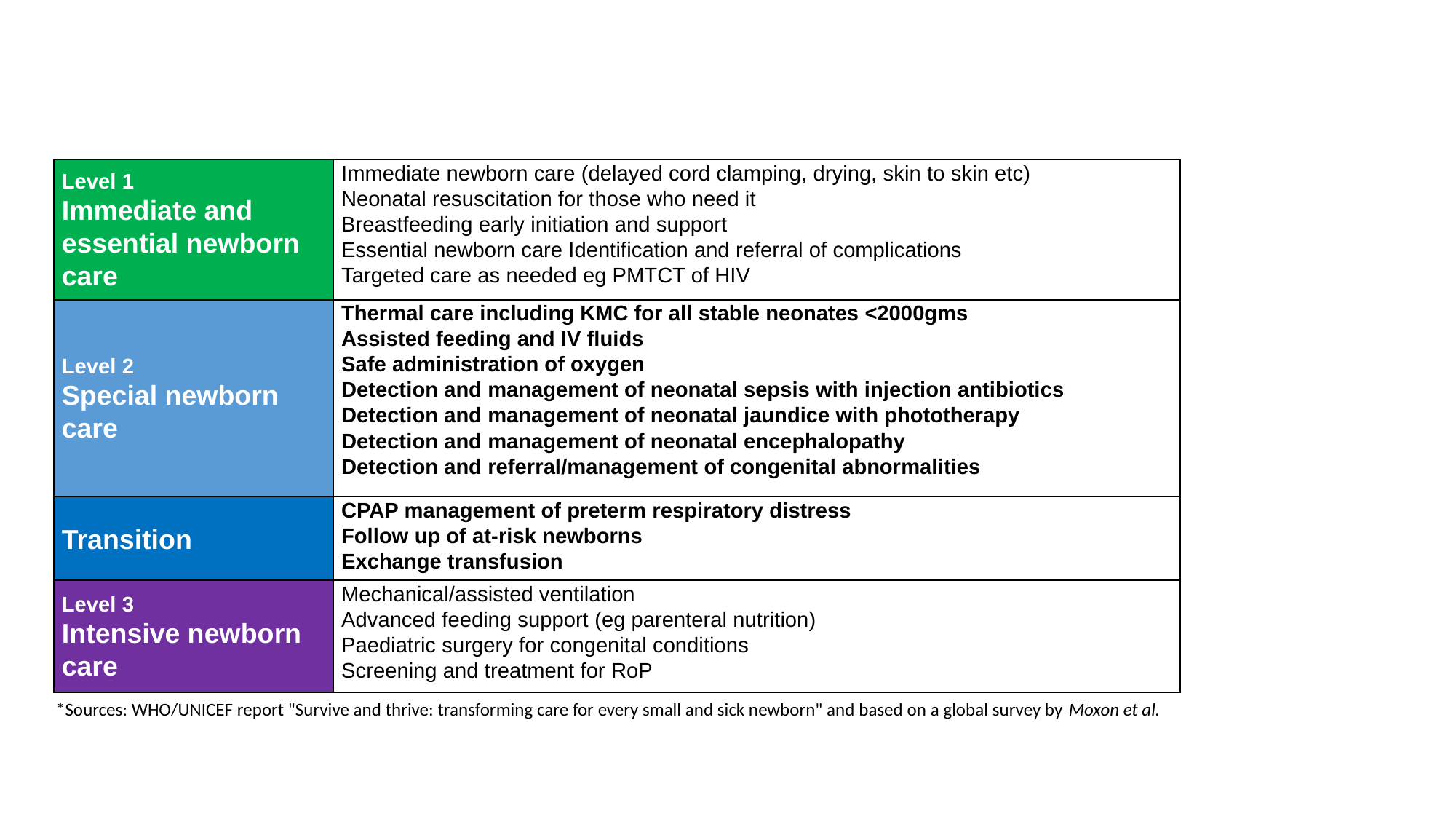

| Level 1 Immediate and essential newborn care | Immediate newborn care (delayed cord clamping, drying, skin to skin etc) Neonatal resuscitation for those who need it Breastfeeding early initiation and support Essential newborn care Identification and referral of complications Targeted care as needed eg PMTCT of HIV |
| --- | --- |
| Level 2 Special newborn care | Thermal care including KMC for all stable neonates <2000gms Assisted feeding and IV fluids Safe administration of oxygen Detection and management of neonatal sepsis with injection antibiotics Detection and management of neonatal jaundice with phototherapy Detection and management of neonatal encephalopathy Detection and referral/management of congenital abnormalities |
| Transition | CPAP management of preterm respiratory distress Follow up of at-risk newborns Exchange transfusion |
| Level 3 Intensive newborn care | Mechanical/assisted ventilation Advanced feeding support (eg parenteral nutrition) Paediatric surgery for congenital conditions Screening and treatment for RoP |
*Sources: WHO/UNICEF report "Survive and thrive: transforming care for every small and sick newborn" and based on a global survey by Moxon et al.
