# Supplementary figures and images for: Quantifying health facility service readiness for small and sick newborn care: comparing standards-based and WHO level-2 + scoring for 64 hospitals implementing with NEST360 in Kenya, Malawi, Nigeria, and Tanzania
Source: BMC Pediatr. 2024 Mar 12;23(Suppl 2):656. doi: 10.1186/s12887-024-04578-5 (PMC10935770; doi:10.1186/s12887-024-04578-5)

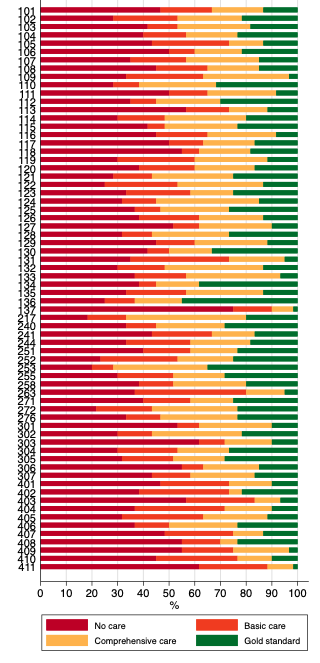

Supplement: Supplementary file 6 — Additional file 6. Stacked bar chart of level-2+ service readiness scoring sub-modules demonstrating overall readiness to provide level-2+ clinical interventions. [file 12887_2024_4578_MOESM6_ESM.png]
